# Exploratory analysis of radiomic as prognostic biomarkers in ^18^F-FDG PET/CT scan in uterine cervical cancer

**DOI:** 10.3389/fmed.2022.1046551

**Published:** 2022-12-02

**Authors:** Nadja Rolim Gonçalves de Alencar, Marcos Antônio Dórea Machado, Felipe Alves Mourato, Mércia Liane de Oliveira, Thauan Fernandes Moraes, Luiz Alberto Reis Mattos Junior, Tien-Man Cabral Chang, Carla Rameri Alexandre Silva de Azevedo, Simone Cristina Soares Brandão

**Affiliations:** ^1^Master of Science Surgery Post-Graduation Program, Federal University of Pernambuco, Recife, Pernambuco, Brazil; ^2^Department of Radiology and Nuclear Medicine, Hospital das Clínicas, Federal University of Pernambuco, Recife, Pernambuco, Brazil; ^3^Department of Radiology, Complexo Hospitalar Universitário Professor Edgard Santos/Universidade Federal da Bahia (UFBA), Salvador, Bahia, Brazil; ^4^Northeast Center for Strategic Technologies, Recife, Pernambuco, Brazil; ^5^Clinical Medicine, Center for Medical Sciences, Federal University of Pernambuco, Recife, Pernambuco, Brazil; ^6^Nuclear Medicine Service, Instituto de Medicina Integrada Fernandes Figueira, Recife, Pernambuco, Brazil; ^7^Department of Oncology, Instituto de Medicina Integrada Fernandes Figueira, Recife, Pernambuco, Brazil

**Keywords:** positron emission tomography, prognosis, uterine cervical neoplasms, 18F-fluorodeoxyglucose, radiomics

## Abstract

**Objective:**

To evaluate the performance of 18F-fluorodeoxyglucose positron emission tomography (^18^F-FDG PET/CT) radiomic features to predict overall survival (OS) in patients with locally advanced uterine cervical carcinoma.

**Methods:**

Longitudinal and retrospective study that evaluated 50 patients with cervical epidermoid carcinoma (clinical stage IB2 to IVA according to FIGO). Segmentation of the 18F-FDG PET/CT tumors was performed using the LIFEx software, generating the radiomic features. We used the Mann–Whitney test to select radiomic features associated with the clinical outcome (death), excluding the features highly correlated with each other with Spearman correlation. Subsequently, ROC curves and a Kaplan–Meier analysis were performed. A *p*-value < 0.05 were considered significant.

**Results:**

The median follow-up was 23.5 months and longer than 24 months in all surviving patients. Independent predictors for OS were found–SUVpeak with an AUC of 0.74, sensitivity of 77.8%, and specificity of 72.7% (*p* = 0.006); and the textural feature gray-level run-length matrix GLRLM_LRLGE, with AUC of 0.74, sensitivity of 72.2%, and specificity of 81.8% (*p* = 0.005). When we used the derived cut-off points from these ROC curves (12.76 for SUVpeak and 0.001 for GLRLM_LRLGE) in a Kaplan–Meier analysis, we can see two different groups (one with an overall survival probability of approximately 90% and the other with 30%). These biomarkers are independent of FIGO staging.

**Conclusion:**

By radiomic ^18^F-FDG PET/CT data analysis, SUVpeak and GLRLM_LRLGE textural feature presented the best performance to predict OS in patients with cervical cancer undergoing chemo-radiotherapy and brachytherapy.

## Introduction

Cervical uterine cancer is an important cause of death in women, especially in regions of low socioeconomic development (1–3). In more advanced stages, fluorine-18-labeled fluorodeoxyglucose positron emission tomography associated with computed tomography (^18^F-FDG PET/CT) is recommended for the adequate evaluation of lymph nodes and distant metastases (4–6).

The standardized uptake value (SUV) of ^18^F-FDG is the most used semi-quantitative variable in ^18^F-FDG PET/CT (7). This value translates the lesion glycolytic metabolism and the higher the value, the more aggressive the tumor (8). Other quantitative metrics extracted from the ^18^F-FDG PET/CT scan are the metabolic tumor volume (MTV), which translates the measure of the tumor volume with a higher metabolism, and the total lesion glycolysis rate (TLG), which is the product of the mean SUV by the lesion MTV (9). These three variables reflect the tumor metabolic load and could help to predict the patient’s prognosis (10, 11).

Radiomic is the extraction of mineable data from medical imaging that has emerged recently (12). It analyzes the lesion phenotype using mathematical formulas that dissect the image, quantifying and characterizing several tumoral features (13–15). Among the numerous variables of the radiomic analysis of ^18^F-FDG PET/CT images, the textural features present a greater correlation with the heterogeneous biological behavior of the tumor. They may serve as predictive markers of overall survival (OS) and therapeutic response (16–18).

Therefore, this paper aims to find radiomic features and metabolic parameters predictive of OS from ^18^F-FDG PET/CT scans of uterine cervical cancer.

## Materials and methods

### Patients and methods

The present study included 50 consecutive patients with histologically confirmed diagnoses of uterine cervical squamous cell carcinoma between 2013 and 2015 (Table 1). The inclusion criteria were: women over 18 years that were undergone pretreatment 18F-FDG PET/CT. All patients received standardized chemotherapy treatment with cisplatin and gemcitabine, with two cycles of neoadjuvant chemotherapy, with subsequent radiotherapy and brachytherapy according to the institutional protocol. The patients were followed up for at least 24 months. The exclusion criteria included ^18^F-FDG PET/CT scans in disagreement with the acquisition, processing, or reconstruction parameters, according to the Image Biomarker Standardization Initiative (IBSI) (19). The selected patients were divided into two groups according to their progression after 24 months of follow-up: group 1, with overall survival of at least 24 months and group 2, deceased due to cancer in the follow-up period. The institutional research ethics committee approved this study. The demographic data and clinical information were obtained from the medical records and included: age, origin, education, smoking status, number of children, and number of sexual partners, in addition to the clinical and imaging staging data (FIGO) (4), and information regarding the treatment.

**TABLE 1:** Clinical and demographic characteristics of the study patients.

Variable	n (%)	%
**N = 47**		
**Age (mean ± SD)**	47 ± 23 years	
**Origin**		
Metropolitan area	26	55.4
Inland cities	21	44.6
**Education**		
Illiterate	17	33.1
0 to 8 years	24	51.0
8 to 12 years	06	12.7
**Smoking**		
Non-smoker	23	48.9
<20 pack-year	04	8.5
>20 pack-year	13	27.6
Ex-smoker for > 5 years	08	14.8
**Number of children**		
1 child	6	12.7
2 children	7	14.8
3 or more children	34	72.3
**Number of sexual partners**		
Up to two partners	12	25.6
Three or two partners	35	74.4
**Tumor size (cm) (mean ± SD)**	5.43 cm (SD 1.49)	
**FIGO staging**		
IB2	02	4.20
II	04	8.0
III	21	44.6
IV	20	42.5

FIGO, international federation of gynecology and obstetrics.

### Protocol of the ^18^F-FDG PET/CT scan

The scans were performed at the nuclear medicine and molecular imaging facility of the *Instituto de Medicina Integral Professor Fernando Figueira* using PET/CT scanner (Siemens Biography 16 channels, Germany), according to the guidelines of the European Society of Nuclear Medicine (20). Patients fasting for at least 4 h and with glycemic levels ≤ 150 mg/dL received 0.14 mCi/kg of ^18^F-FDG intravenously. Approximately 60 min after the administration of ^18^F-FDG, images were obtained from the skull to the thigh root. All the patients received 20 mg of furosemide after the first imaging; additionally, 120 min after the radiopharmaceutical injection, they returned to the scanner for late imaging of the pelvis. The acquisition parameters of the initial images were analyzed, with a reconstruction diameter of 500 mm, tube voltage of 130 kV, current of 75–310 mAs, and thickness of 3 mm. The images were reconstructed with 3D OSEM mode (four iterations and eight subsets) in a 4.07 × 4.07 × 5.00 mm^3^ matrix.

### Radiomic analysis

#### Segmentation

We use the free access multiplatform Local Image Features Extraction (LIFEx) software (V6.30—Inserm, Orsey, France) (21), as can be seen in Supplementary Figure 1. Initially, a semi-automatic segmentation of the uterine cervical lesion was performed (whole-body image only), identified by the ^18^F-FDG uptake on the CT fusion image, and manually outlined with a 3D design tool. Subsequently, the software selected the area of highest uptake, considering a fixed threshold of 40% of the standard uptake value (SUV) of the ROI volume (VOI), a method validated for cervical uterine neoplasms (22, 23). Notably, the details regarding the computation parameters and formulas are described at www.lifexsoft.org (21). A radiologist specialized in the female pelvis and supervised by a nuclear medicine specialist, both with 20 years of experience, did the segmentations for all patients.

#### Extraction

For each selected volume, a massive extraction of numerical data was performed by LIFEx, using 4 × 4 × 4 resizing, 0.25 fixed number width (FBW) intensity discretization method and histogram redefinition, obtaining 50 tumor features. These features were divided into categories, including: first-order statistics derived from the voxel intensity histogram (shape, volume, and histogram), and conventional indices (SUVpeak, SUVmean, SUVmax, MTV, and TLG); second-order statistics, including features based on the gray-level co-occurrence matrix (GLCM), gray-level run-length matrix (GLRLM), neighboring gray-level dependence matrix (NGLDM), and gray-level zone length matrix (GLZLM) (12, 21, 24).

#### Selection of radiomic features

Initially, searching for clinically significant markers associated with OS, we performed an independent sample test with the Mann–Whitney to assess the distribution for each feature in the two groups, including those with a *p*-value < 0.05, to subsequent analysis.

After that, the data were submitted to dimension reduction through rank correlation with Spearman’s coefficient, evaluating each pair of features. Later, we found which markers correlated with each other, excluding redundant markers using a correlation matrix and selecting those with a pre-established hypothetical *rho* lower than 0.85.

### Statistical analysis

The absolute and relative frequency described categorical variables in percentage. Continuous variables with a normal distribution were analyzed by the mean and standard deviation; while non-parametric variables were analyzed by the median, maximum and minimum values, and interquartile range (IQR). For comparison between variables, we used the Mann–Whitney *U* test. We determined the cut-off points for variables with a *p-*value < 0.05 and the distinction between groups by ROC curves (DeLong methodology).

For prognostic evaluation, we correlated the selected radiomic features with the OS. Kaplan–Meier survival curves were constructed, with cut-off points obtained by the ROC curve for each variable, using the MedCalc software (MedCalc Software Ltd, Ostend, Belgium; https://www.medcalc.org; 2022). *P*-values lower than 0.05 were considered statistically significant.

## Results

### Clinical and demographic characteristics

The sample was initially composed of 50 consecutive patients. Three patients were excluded: one whose pretreatment baseline scan was unavailable and two other scans with divergence in the acquisition parameters (disagreement with IBSI standards).

Therefore, 47 patients were eligible for this study, with a mean age of 47 ± 23 years and ranging from 24 to 70 years. The majority of the patients had a low level of education, with reports of multiple sexual partners. Approximately 87% of patients presented with advanced stages of the disease (FIGO III and IV) (Table 1). Advanced stages of FIGO were correlated with lower overall survival (Supplementary Figure 2).

The median follow-up was 23.5 months (range: 3.73–39 months), with all surviving patients being followed up for at least 24 months. Of a total of 47 patients, 36 (77%) were alive at the end of 24 months (group 1) and 11 (23%) patients had died due to the disease (group 2).

### Metabolic biomarkers and textural radiomic features

The data were extracted from 47 VOIs. We selected the features with discriminatory power for the selected outcome: three metabolic parameters–SUVmax (*p* = 0.02), SUVmean (*p* = 0.02), and SUVpeak (*p* = 0.01); and 13 textural markers–five markers from the GLZLM matrix (GLZLM_SZE, GLZLM_LGZE, GLZLM_HGZE, GLZLM_SZLGE, and GLZLM_SZHGE); six markers from the GLRLM matrix (GLRLM_LGRE, GLRLM_HGRE, GLRLM_SRLGE, GLRLM_SRHGE, GLRLM_LRLGE, and GLRLM_LRHGE); and two markers from the GLCM matrix (GLCM_Contrast variance and GLCM_Dissimilarity).

Among the metabolic parameters, the SUVpeak showed the best performance to differentiate the groups. The SUVpeak median in group 1 was 10.89 (IQR 7.60–12.69), while in group 2 was 13.87 (IQR 12.17–14.14), *p* = 0.01. The best cut-off point value (ROC curve analysis) was 12.76 with an AUC of 0.74, a sensitivity of 77.8%, and a specificity of 72.7%, *p* = 0.006.

The SUVmax median value in group 1 was 12.83 (IQR 9.09–14.90) vs. 15.98 in group 2 (IQR 13.52–19.09), *p* = 0.02. The best cut-off point was 14.32, AUC = 0.68, sensitivity = 72.3%, and specificity = 72.7% for the cut-off point of 14.32 (*p* = 0.012). The SUVmean median value in group 1 was 7.68 (IQR 9.09–14.90) vs. 9.88 in group 2 (IQR 8.88–10.92), *p* = 0.02. It presented an AUC of 0.68, sensitivity of 72.3%, and specificity of 72.7% for a cut-off point of 8.8 (*p* = 0.01).

The other conventional metabolic metrics were not significant. The median MTV in group 1 was 31.9 (IQR: 18.5–51.0) vs. 37.8 (IQR: 24.6–72.4) in group 2 (*p* = 0.49). The median TLG in group 1 was 295.9 (IQR: 100.7–403.7) vs. 320.3 (IQR: 253.2–465.7) in group 2, *p* = 0.33.

Aiming for the redundancy feature reduction, we used the Spearman rank correlation for each of these 13 attributes. Three of them showed a rho value lower than 0.85: GLRLM_LGRE, GLRLM_SRLGE, and GLRLM_LRLGE. When we compared the AUC of these three indices, the GLRLM_LRLGE textural feature presented a little better performance. The GLRLM_LRLGE median in group 1 was 1.2 × 10^–3^ (IQR: 8 × 10^–4^–32 × 10^–3^) vs. 7.7 × 10^–3^ in group 2 (IQR 6 × 10^–4^–9 × 10^–3^, *p* = 0.017). The best cut-off point value was 1 × 10^–3^ (AUC: 0.74; sensitivity: 72.2%; specificity: 81.8%, *p* = 0.005).

**FIGURE 1:**
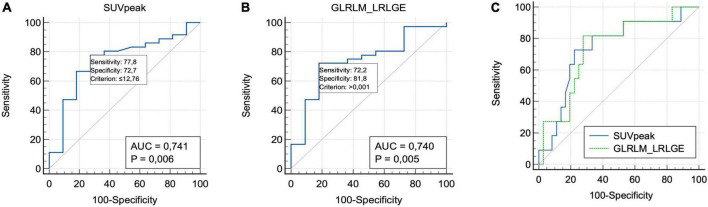
Receiver operating characteristic curve analyses of SUVpeak **(A)**, GLRLM_LRLGE **(B)**, and both **(C)** value for predicting overall survival.

For GLRLM_LGRE, the group 1 median value was 1.2 × 10^–3^ (IQR: 7 × 10^–4^–2.4 × 10^–3^), and in group 2 was 7 × 10^–4^ (IQR: 5 × 10^–4^ to 8 × 10^–4^, *p* = 0.01). It presented an AUC of 0.73, sensitivity of 81.8%, and specificity of 72.2% for a cut-off point of 9 × 10^–4^ (*p* = 0.006). For GLRLM_SRLGE, the group 1 median value was 1.2 × 10^–3^ (IQR: 7 × 10^–4^–2.3 × 10^–3^), and in group 2 was 7 × 10^–4^ (IQR: 5 × 10^–4^–8 × 10^–4^, *p* = 0.01). The AUC was 0.73, sensitivity of 81.8%, and specificity of 72.2% for a cut-off point of 8 × 10^–4^ (*p* = 0.006) (Figure 1). More information at Table 2.

**TABLE 2:** Results of independent-samples Mann–Whitney test analysis parameters 18F-FDG PET/CT for cervical cancer predicting overall survival.

	Group 1		Group 2		
	Median	Range	Median	Range	*P*
**Image-based parameters**					
SUV mean	7.68	9.09–14.90	9.88	8.88–10.92	0.02
SUV peak	10.89	7.60–12.69	13.87	12.17–14.14	0.01
SUV max	12.83	9.09–14.90	15.98	13.52–19.09	0.02
TLG (mL)	295.9	100.7–403.7	320.3	253.2–465.7	0.33
MTV (mL)	31.9	18.5–51.0	31.9	24.6–72.4	0.49
**Texture parameters**					
GLRLM_LGRE	1.2 × 10^–3^	7 × 10^–4^–2.4 × 10^–3^	7 × 10^–4^	5 × 10^–4^–8 × 10^4^	0.01
GLRLM_SRLGE	1.2 × 10^–3^	7 × 10^–4^–2.3. 10^–3^	7 × 10^–4^	5 × 10^–4^–8 × 10^4^	0.01
GLRLM_LRLGE	1.2 × 10^–3^	8 × 10^–4^–3.2 × 10^–3^	7.7 × 10^–3^	6 × 10^–4^–9 × 10^4^	0.01

^18^F-FDG PET/CT, ^18^fluorodeoxyglucose positron emission tomography; Group 1, survivors; Group 2, dieded; SUV, standardized uptake values; SUVmax, maximum standardized uptake value; SUVmean, mean standardized uptake value; SUVpeak, the peak of SUV in 1 mL; TLG, total lesion glycolysis; MTV, metabolic tumor volume; GLRLM, Gray level run length matrix; LGRE, low gray-level runs emphasis; SRLGE, short runs low gray-level emphasis; LRLGE, long runs gray-level emphasis.

### Correlations between conventional parameters ^18^F-FDG PET/CT and textural features

The SUVpeak showed negative correlations with the GLRLM matrix. GLRLM_LRLGE (*r* = −0.890, *p* < 0.01). The SUVmax also showed a negative correlation with GLRLM_LRLGE (*r* = 0.764, *p* < 0.01).

Both SUVpeak and GLRLM_LRLGE were not correlated with FIGO staging (Supplementary Table 1).

### Kaplan–Meier survival analysis

GLRLM_LRLGE showed a significant correlation with the OS (*p* = 0.003). Patients who died presented a GLRLM_LRLGE value lower than the cut-off point, with a shorter survival time: median of 708 days (CI: 505.0–734.0). A risk ratio of 10.8 (CI: 3.0–39.1) was observed.

**FIGURE 2:**
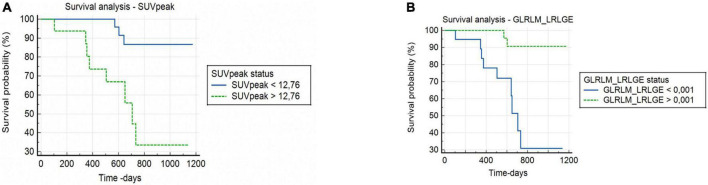
Kaplan–Meier survival curve of overall survival in patients with cervical cancer. **(A)** High SUVpeak value (>12.76) and low SUVpeak value (<12.76). **(B)** High GLRLM_LRLGE value (>1.10^– 3^) and low GLRLM_LRLGE value (<1.10^– 3^).

SUVpeak showed a significant correlation with the OS (*p* = 0.006). Patients who died presented a higher SUVpeak value, with a shorter survival time: a median of 706 days (CI: 374.0–734.0). A risk ratio of 10.5 (CI: 2.7–40.3) was observed (Figure 2).

## Discussion

This study demonstrated the prognostic association between radiomic biomarkers of primary uterine cervical cancer lesions at ^18^F-FDG-PET/CT and overall survival. Among the evaluated metabolic parameters, SUVpeak showed the best discriminatory power; and among all the selected radiomic textural features, the GLRLM_LRLGE presented the best predictive performance. Moreover, SUVpeak and GLRLM_LRLGE demonstrated a greater correlation with OS compared with clinical and other more conventional ^18^F-FDG-PET/CT parameters, including MTV and TLG. These data reinforce the importance of metabolic radiomic evaluation in cervical uterine tumor staging.

Cervical cancer accounts for high morbidity and mortality in patients of productive and reproductive age worldwide, especially in vulnerable populations (25). The staging of this neoplasm is based on FIGO classification, which includes characteristics of the primary lesion, and lymph node or distant dissemination (4–6). However, FIGO classification presented a low accuracy in predicting therapy response and survival, especially among patients with advanced-stage cancer disease (4).

The search for non-invasive and robust prognostic biomarkers can improve the predictive power of therapy response. Radiomic is considered a promising analysis tool in precision medicine (26, 27). Some studies have also reported the use of this technology in cervical tumor cases, based on several imaging methods, especially magnetic resonance imaging and ^18^F-FDG-PET/CT (28). Usually, these studies aim to evaluate several aspects of the tumor, covering most frequently lymph node invasion (29–31), prognosis (28, 32), and therapeutic response (33); followed by histological grade (34–36), staging (37), and lymphovascular space invasion (29).

Standardized uptake value represents a semi-quantitative metric of ^18^F-FDG-PET/CT with prognostic ability, including OS evaluation of patients with uterine cervix tumors (10, 33). All metrics of SUV are correlated with each other, providing information on the tumor metabolic activity. SUVpeak is reported as more robust and reproducible than SUVmax and SUVmean, although it is not widely disseminated in clinical practice (7). Studies report better performance of SUVpeak to demonstrate the aggressiveness of early-stage cervical tumors compared to SUVmax, maybe because SUVpeak measures several voxels in a more metabolically active spherical VOI of the lesion (7, 38).

The SUVpeak in our study presents a cut-off value similar to those described in other studies. Schernberg et al. (39) analyzed locally advanced disease treated with definitive chemoradiation and demonstrated that a high SUVpeak value was superior in predicting the OS and local recurrence, when compared with other ^18^F-FDG-PET/CT parameters, like MTV and TLG. Other studies also evaluated early-stage cervical cancer, in which a low SUVpeak was significantly correlated with high progression-free survival (40).

A systematic review by Piñeiro-Fiel et al. evaluated the radiomics of ^18^F-FDG-PET/CT in several neoplasms. Gynecological cancers were among the four most studied types, with 19 publications in a total of 741 studies. Of these 19 publications, cervical uterine cancer accounted for the largest number of publications (74%), followed by endometrial cancer (16%). As in our study, the textural features were correlated with the conventional metrics of ^18^F-FDG-PET/CT, including SUV. In the analysis of gynecological cancers, the texture matrices that presented higher significance were GLCM, GLRLM, and GLZSM (41).

We showed that GLRLM_LRLGE could perform well in predicting OS in patients with advanced cervical cancer. The radiomic matrix GLRLM conceptually relates to the intensity of the gray level of pixels in an image, in a given direction, and LRLGE represents the distribution of long stretches with a high or low gray level, being an indicator of the uniformity of the homogeneous distribution of FDG uptake (42). GLRLM_LRLGE is a potential biomarker in other neoplasms too, as it can discriminate benign from malignant renal tumors (42), and can be used to assess recurrence in rectal cancer (43).

Additionally, some studies demonstrated a significant correlation between the GLRLM matrix 18F-FDG-PET/CT textural markers (LGRE, SRLGE, and LRLGE) and RNA-level immunological biomarkers of PD-L1 (programmed death ligand 1) in lung cancer (44). PD-L1 protein expression is also a predictive biomarker in uterine cervical cancer (45). Subsequently, we could find a possible intercorrelation between these textural markers (GLRLM_LRLGE) and PD-L1 expression, representing an important prognostic and selection factor for immunotherapy. This hypothesis may be evaluated in future prospective studies.

GLRLM_LRLGE possibly shows a relationship with tumor necrosis, as it assesses the homogeneity of ^18^F-FDG uptake, and its highest value is documented in benign homogeneous lesions (42). On the other hand, several studies demonstrate a direct relationship between PD-L1 and tumor necrosis factor (TNF alpha) in oncologic diseases, including findings in which TNF alpha produced by adipocytes positively regulates PD-L1 (46). Based on these findings, we can assume that the textural factor GLRLM_LRLGE also correlates with TNF alpha.

However, this study has many limitations. It is a single-center study with a low number of patients and a retrospective analysis. However, the sample was derived from a clinical trial (47), with a relatively homogeneous and controlled group of patients with a good clinical follow-up. Additionally, the ^18^F-FDG-PET/CT pretreatment images were reevaluated in order to collect new data regarding radiomic characteristics in the primary lesions. Moreover, we analyzed only the scans with a protocol following the parameters established by the IBSI. We also do not perform multiple correction tests in our data, mainly because of the low number of included patients.

In conclusion, in patients with advanced cervical tumors, this study investigated and identified two biomarkers with better prognostic performance (SUVpeak and GLRLM_LRLGE). These features denote metabolism and intratumoral textural homogeneity, respectively. In the future, the SUVpeak and GLRLM_LRLGE have the potential to be incorporated into clinical practice, helping to identify patients with a higher risk of death.

## Data availability statement

The raw data supporting the conclusions of this article will be made available by the authors, without undue reservation.

## Ethics statement

The studies involving human participants were reviewed and approved by Cômite de Ética em Pesquisa do IMIP. Written informed consent for participation was not required for this study in accordance with the national legislation and the institutional requirements.

## Author contributions

NA, SB, and MM conceptualized and designed the study. T-MC and CA were acquired the data. NA and TM identified and marked the lesions. MM and FM inferred results, implemented the methods, and analyzed and interpreted the data. NA, MO, and SB drafted the manuscript and wrote the first draft of the manuscript. LM, FM, SB, and MO critically revised the manuscript and provided supervision, support, conceptualization, and guidance throughout the project. All authors contributed to the article and approved the submitted version.
