# Supplementary material for: Exploratory analysis of radiomic as prognostic biomarkers in 18F-FDG PET/CT scan in uterine cervical cancer
Source: Front Med (Lausanne). 2022 Dec 2;9:1046551. doi: 10.3389/fmed.2022.1046551 (PMC9769204; doi:10.3389/fmed.2022.1046551)
Supplement: Supplementary file 1 [file Data_Sheet_1.docx]

# SUPPLEMENTARY FILE

# ALENCAR ET AL.

Supplementary Figure 1: Clinical imagining showing LifeX utilization.


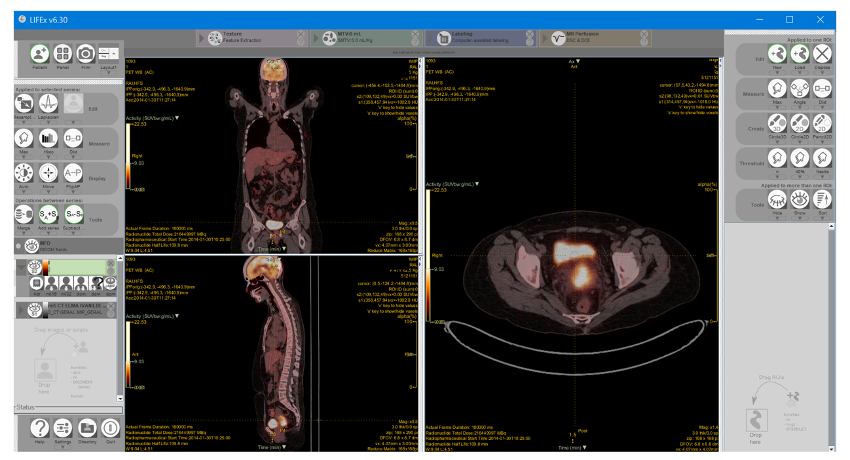


Supplementary Figure 2: Comparison between FIGO groups by Overall survival


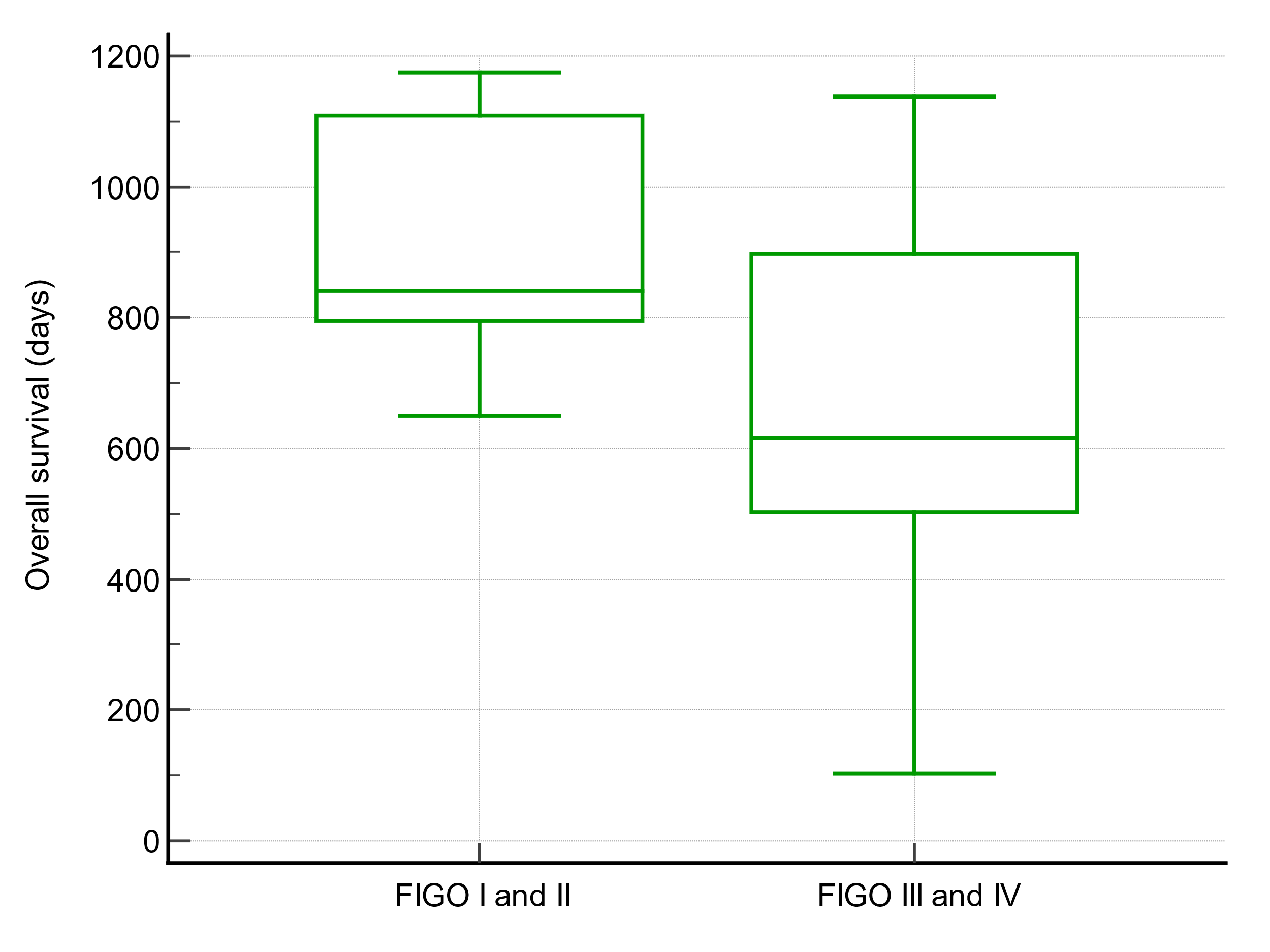


Legend Supplementary Figure 1: Box-and-Whisper graph comparing overall survival between FIGO groups using Mann-Whitney test (p = 0.02).

Supplementary table 1: GLRLM_LRLGE radiomic feature and SUVpeak characteristics among different FIGO groups

| Variable | FIGO I and II | | | FIGO III and IV | | |  |
| --- | --- | --- | --- | --- | --- | --- | --- |
|  | n | Median | Average Rank | n | Median | Average Rank | p-value |
| GLRLM_LRLGE | 6 | 0,0025 | 32,1667 | 41 | 0,001091 | 22,8049 | 0,1183 |
| SUVpeak | 6 | 7,9700 | 18,8333 | 41 | 11,7600 | 24,7561 | 0,3230 |

^a^ Mann-Whitney test
